# Genomic characteristics of driver genes in Chinese patients with non‐small cell lung cancer

**DOI:** 10.1111/1759-7714.13757

**Published:** 2020-12-09

**Authors:** Xiaoyan Si, Ruili Pan, Shaohua Ma, Lin Li, Li Liang, Ping Zhang, Yuping Chu, Hanping Wang, Mengzhao Wang, Xiaotong Zhang, Li Zhang

**Affiliations:** ^1^ Department of Pulmonary and Critical Care Medicine Peking Union Medical College Hospital Beijing China; ^2^ Department of Thoracic Surgery Peking University Third Hospital Beijing China; ^3^ Department of Oncology Beijing Hospital Beijing China; ^4^ Department of Cancer Chemotherapy and Radiation Peking University Third Hospital Beijing China; ^5^ Department of Oncology Beijing Chaoyang Hospital Beijing China

**Keywords:** Driver gene, next‐generation sequencing, non‐small cell lung cancer

## Abstract

**Background:**

The aim of this study was to determine the demographic profile of driver gene alterations, especially low‐frequency gene alterations in Chinese patients with non‐small cell lung cancer (NSCLC).

**Methods:**

A total of 7395 Chinese patients with NSCLC were enrolled in the study. Next‐generation sequencing (NGS) was performed on formalin‐fixed paraffin‐embedded specimens collected via either surgical resection or biopsy.

**Results:**

The frequent genomic alterations found in the study were *EGFR* mutations (51.7%), *KRAS* mutations (13.1%), *MET* alterations (5.6%; 3.2% copy number gains and 0.5% exon 14 skipping mutation), *HER2* alterations (7.0%; 2.0% copy number gains and 5.4% mutations), *ALK* alterations (7.2%; 3.9% rearrangements), *RET* rearrangements (1.4%), *ROS1* rearrangements (0.9%), and *NTRK* rearrangements (0.6%). The *EGFR* mutation rate was found to be significantly higher in women than in men (69.1% vs. 38.5%, *P* < 0.001), while the *KRAS* mutation (17.5% vs. 7.3%, *P* < 0.001) and *MET* alteration rates (6.5% vs. 4.5%, *P* < 0.001) were significantly higher in men than in women. The *EGFR* mutation rate tended to decrease with age in the group aged >40 years, while the *KRAS* mutation rate tended to increase with age. The *HER2* mutation (13.9% vs*.* 6.7%, *P* < 0.001) and *ALK* alteration rates (14.3% vs. 6.9%, *P* < 0.001) were significantly higher in the group aged <40 years than in groups aged 40 years or older.

**Conclusions:**

The frequency of different driver genes was diverse in different age‐gender groups, and the results of this study may assist clinicians in clinical decision‐making and the development of public healthcare strategies in the future.

**Key points:**

**Significant findings of the study:**

- This study demonstrated that the frequency of different driver genes was diverse in different age‐gender groups.

**What this study adds**

- It may enable clinicians to make clinical decisions, and assist government, pharmaceutical researchers and insurance companies develop public healthcare strategies.

## Graphical abstract

This study demonstrated that the frequency of different driver genes was diverse in different age‐gender groups, and may assist clinicians in clinical decision‐making, and assist government, pharmaceutical researchers and insurance companies in the development of public healthcare strategies in the future.

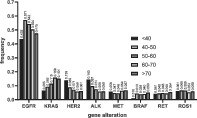

## Introduction

Lung cancer is the most commonly reported cancer and leading cause of cancer death in China.^1^ Non‐small cell lung cancer (NSCLC) accounts for approximately 85% of patients with lung cancer.^2^ Targeted therapies have dramatically changed the treatment modalities for NSCLC. The National Comprehensive Cancer Network (NCCN) guideline for NSCLC (Version 1.2020) recommends targeted treatment for *EGFR*, *ALK*, *ROS1*, *BRAF*, *NTRK*, *RET*, *HER2*, *MET* amplification and exon 14 skipping mutation. It is well known that patients of East Asian ethnicity have a different prevalence of oncogenic mutations.^3^ It is therefore essential that the demographic profile of driver genes in East Asian patients with NSCLC is determined. *EGFR* mutation has been widely and well researched, while alterations of *BRAF*, *HER2*, *MET*, *ROS1*, *RET*, and *NTRK* have not been previously well described due to their low frequency. Here, we studied the demographic characteristics of driver gene alterations in Chinese patients with NSCLC identified by next‐generation sequencing (NGS). The large sample size of the study made it possible to describe the low‐frequency gene alterations.

## Methods

### Patients and samples

A total of 7395 Chinese patients were enrolled in the study. Formalin‐fixed paraffin‐embedded (FFPE) specimens were analyzed from patients with NSCLC who underwent either surgical resection or biopsy from 1 January 2018 to 1 October 2019. In order to ensure the quality of DNA extraction, FFPE specimens retrieved within one year were selected, and the specimens were reviewed by experienced pathologists. All the patients involved provided their written informed consent. The study was approved by the Institutional Review Board of Peking Union Medical College Hospital (S‐K1264).

### DNA extraction and sequencing library preparation

NGS was performed in the CAP‐accredited laboratory. The tumor content of all samples was confirmed to be at least 10% by pathologists. FFPE sections were deparaffinized with xylene, from which genomic DNA was extracted using the BLACK PREP FFPE DNA kit according to the manufacturer's protocol. The quantity and quality of the extracted DNA were evaluated using a Qubit 3.0 fluorometer and Bioanalyzer 2100 (Agilent Technologies), respectively (Thermo Fisher Scientific). The DNA was fragmented using a Covaris M220 sonication system to obtain 200 bp fragments and purified using Agencourt AMPure XP beads (Beckman Coulter). Library preparations of the fragmented DNA were performed using the KAPA Hyper Prep Kit (KAPA Biosystems), following the manufacturer's protocol. Libraries with different indices were pooled for Hypercap Target Enrichment Kit, and a customized enrichment panel (Roche) covering the exonic regions of 290 genes and the introns of 26 fusion genes. The captured library was further amplified using Illumina p5 (5′ AAT GAT ACG GCG ACC ACC GA 3′) and p7 (5’ CAA GCA GAA GAC GGC ATA CGA GAT 3′) primers in the KAPA Hifi HotStart ReadyMix (KAPA Biosystems), and purified with Agencourt AMPure XP beads. Sequencing libraries were quantified by Bioanalyzer 2100 (Agilent Technologies). The final libraries were sequenced on an Illumina Novaseq 6000 platform to a mean coverage depth of at least 250×, following the manufacturer's instructions.

### Bioinformatic analysis

Genomic alterations, including single nucleotide variants (SNVs), short and long insertions/deletions (indels), copy number variations (CNVs), and gene fusions, were subjected to advanced analysis. First, reads were aligned to human genome reference sequence (hg19) by BWA (0.7.17), and duplication reads were removed using Novosort (3.08.00). Second, SNVs and short indels were identified by VarScan (2.4.2) after quality recalibration and realignment using a genome analysis toolkit (GATK) and in‐house pipeline. Short indels were then calibrated using the results from Pindel. A customized algorithm ctCNV was developed to identify and filter CNV. The thresholds of copy number ≥ 2.5 and ≤ 1.5 were used to categorize altered regions as CNV gains (amplification) and copy number losses (deletions). FusionMap (8.0.2.32) was used to detect gene fusion. Gene fusions were required to have at least two support reads with a background *P*‐value under 0.05. More importantly, reliable somatic alterations were detected in the raw data by comparison with matched blood control samples. At a minimum, five reads and minimum variant allele frequency of 1% were required to support alternative calling.

### Statistical analysis

Patients were grouped by age as <40 years, 40–49 years, 50–59 years, 60–69 years, and 70 years or older. Statistical analysis was performed by R language. The χ2‐test was used to analyze the associations of mutational status with gender and age groups. A two‐tailed *P*‐value of <0.05 was considered statistically significant.

## Results

### Demographic characteristics

The demographic characteristics are shown in Table 1. With regard to the histological subtype, 5378 cases (72.7%) were lung adenocarcinoma, 855 (11.6%) squamous cell carcinoma, and 1162 (15.7%) NSCLC‐not otherwise specified. The median age of patients was 60 years (range: 8–94), and 56.6% of patients were male.

**Table 1:** Genomic profiling of patients in the study

	NSCLC^†^ (*n* = 7395)	LAD^‡^ (*n* = 5378)	LSCC^§^ (*n* = 855)
Median age (range)	60 (8–94)	59 (8–94)	63 (28–93)
Gender (M/F)	4189/3206	2678/2700	770/85
<40 (M/F: 127/167)	294/4.0%	242/5.0%	10/1.2%
40–49 (M/F:462/567)	1029/13.9%	801/14.9%	65/7.6%
50–59 (M/F: 1200/1082)	2282/30.9%	1716/31.9%	235/27.5%
60–69 (M/F: 1629/990)	2619/35.4%	1849/34.4%	360/42.1%
≥70 (M/F: 771/400)	1171/15.8%	770/14.3%	185/21.6%
Smoking history			
Smoking	2670	1725	469
Non‐smoking	3837	3006	291
Unknown	888	647	95
*EGFR* mutations (%)	3821/51.7%	3177/59.1%	156/18.2%
*KRAS* mutations (%)	966/13.1%	757/14.1%	63/7.4%
*HER2* alterations (%)	517/7.0%	400/7.4%	45/5.3%
*HER2* CNGs ¶ (%)	149/2.0%	98/1.8%	23/2.7%
*ALK* alterations (%)	531/7.2%	389/7.2%	59/6.9%
*MET* alterations (%)	414/5.6%	304/5.6%	46/5.4%
*MET* CNGs ¶ (%)	240/3.2%	184/3.4%	23/2.7%
*BRAF* alterations (%)	298/4.0%	225/4.2%	24/2.8%
*ALK* rearrangements (%)	286/3.9%	233/4.3%	11/1.2%
*RET* rearrangements (%)	103/1.4%	95/1.8%	0/0.0%
*ROS1* rearrangements (%)	67/0.90%	55/1.02%	2/0.2%
*NTRK* rearrangements (%)	44/0.59%	33/0.61%	4/0.5%

^†^ Non‐small cell lung cancer.

^‡^ Lung adenocarcinoma.

^§^ Lung squamous cell carcinoma.

^¶^ Copy number gains.

### Driver gene alterations

The genomic alteration spectra are shown in Table 1. Frequent genomic alterations found were *EGFR* mutations (51.7%), *KRAS* mutations (13.1%), *MET* alterations (5.6%; 3.2% copy number gains and 0.5% exon 14 skipping), *HER2* alterations (7.0%; 2.0% copy number gains and 5.4% mutations), *ALK* alterations (7.2%; 3.9% rearrangements), *RET* rearrangements (1.4%), *ROS1* rearrangements (0.9%), and *NTRK* rearrangements (0.6%). A total of 5069 (68.5%) patients harbored driver genes. The National Comprehensive Cancer Network (NCCN) guidelines for NSCLC recommend that biomarker testing should include *EGFR* mutation, *ALK* rearrangement, *ROS1* rearrangement, *NTRK* gene fusion, *MET* amplification, *MET* exon 14 skipping mutation, *RET* rearrangement, and *HER2* mutation (Table 2).

**Table 2:** Genomic alteration spectra of non‐small cell lung cancer (NSCLC)

Genomic alteration (N)	Alteration distribution (N)
*EGFR* mutations (3821)	Exon 18(217), exon 19 (1572), exon 20 (439), exon 21(1757)
*KRAS* mutations (600)	Exon 2(443), exon 3 (63), exon 4 (32)
*MET* alterations (414)	copy number gain (240), exon 14 skipping (36)
*HER2* mutations (397)	Exon 18 (3), exon 19 (7), exon 20 (227), exon 21(7)
*BRAF* alterations (298)	V600E (75), K601E (32), G469A (19), D22N (13), G469V (8), D594G (8), G466V (6), N588I (6), D594N (6); copy number gain (17); CDC27‐BRAF (2), SND1‐BRAF (2)
*ALK* rearrangements (286)	EML4‐ALK (272), HIP1‐ALK (5), KLC1‐ALK (2), STRN‐ALK (2)
*RET* rearrangements (103)	KIF5B‐RET (81), CCDC6‐RET (16)
*ROS1* rearrangements (67)	CD74‐ROS1(28), SDC4‐ROS1 (12), SLC34A2‐ROS1 (11), EZR‐ROS1 (10), TPM3‐ROS1 (4), ERC1‐ROS1 (2)
*NTRK* rearrangements (44)	AGBL4‐NTRK (19), ETV6‐NTRK (13), VCL‐NTRK (6), TRIM24‐NTRK (3)

*EGFR* was the most frequently determined mutated gene in Chinese patients with NSCLC. Exon 21 L858R (*n* = 1645) and exon 19 deletions (*n* = 1526) accounted for 82.9% of all detected *EGFR* mutations. Other *EGFR* mutations included *T790M* (*n* = 178, 4.6%), exon 20 insertion (*n* = 148, 3.8%), *G719X* (*n* = 143, 3.7%), *L861Q* (*n* = 78, 2.0%), *S768I* (*n* = 71, 1.8%), *E709X* (*n* = 38, 1.0%), and *V834L* (*n* = 22, 0.6%). A total of 678 patients (17.7% of patients with *EGFR* mutations) were identified as having complex *EGFR* mutations. The most common complex mutation was *T790M* with another mutation (*n* = 172, 4.5% of *EGFR* mutations). Table 3 identifies the 10 complex *EGFR* mutation types found with the highest frequency.

**Table 3:** Complex *EGFR* mutations identified in non‐small lung cancer (NSCLC)

Complex mutation type	Number	Percentage of *EGFR* mutations (*n* = 3821)
Exon 19 deletion + *T790M*	91	2.4%
*L858R* + *T790M*	75	2.0%
*G719X* + *E709X*	30	0.8%
Exon 19 deletion + *L858R*	28	0.7%
*G719X* + *S768I*	27	0.7%
*L858R* + *E709X*	18	0.5%
*L858R* + *V834L*	17	0.4%
*G719X* + *L861X*	13	0.3%
*L858R* + *S768I*	11	0.3%
Exon 19 deletion + *K754X*	10	0.3%

*HER2* alterations were identified in 517 patients, including *HER2* copy number gains (CNGs) in 150 patients, *HER2* mutations in 397 patients, and both *HER2* CNGs and mutations in 29 patients. *HER2* mutations were distributed in ligand binding domain 1 (*n* = 6), cysteine‐rich domain (*n* = 28), ligand binding domain 2 (*n* = 7), growth factor receptor domain (*n* = 52), transmembrane domain (*n* = 17), and tyrosine kinase domain (*n* = 243). The most frequent type of *HER2* alteration were exon 20 mutations in the kinase domain (*n* = 228). Y772_G775dupYVMA was the most common exon 20 variant (*n* = 144), followed by E770delinsEAYVM (*n* = 32), G776delinsVC (*n* = 20), G778_P780dupGSP (*n* = 9), and G776delinsVV (*n* = 8). S310F mutations in the *HER2* extracellular region were identified in 13 patients.

The most common subtypes of *ALK*, *RET*, *ROS1* and *NTRK* rearrangement were EML4‐ALK, KIF5B‐RET, CD74‐ROS1, and AGBL4‐NTRK, respectively.

### Correlations between genomic alterations and gender

**Figure 1:**
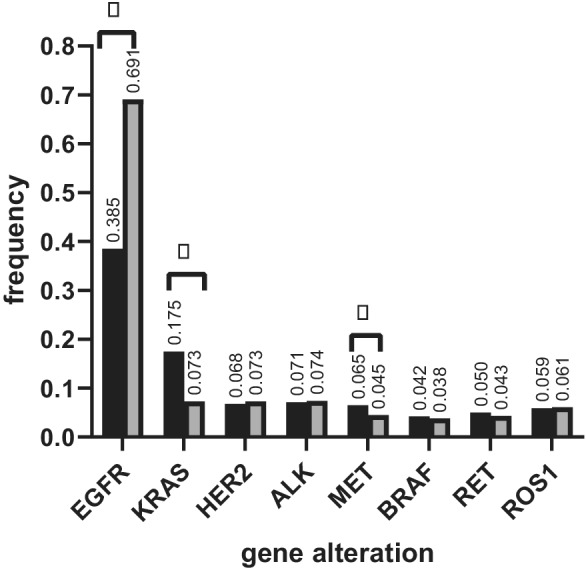
Comparison of frequency of selected gene mutations in Chinese patients with non‐small cell lung cancer (NSCLC) between men and women. *EGFR*, *EGFR* mutations; *KRAS*, *KRAS* mutations; *HER2*, *HER2* alterations; *ALK*, *ALK* alterations; *MET*, *MET* alterations; *BRAF*, *BRAF* alterations; *RET, RET* rearrangements; *ROS1*, *ROS1* rearrangements; *, significant difference. (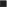) Male, (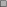) Female.

Correlations of genotype with gender are shown in Fig 1. The *EGFR* mutation rate was found to be significantly higher in women than in men (69.1% vs. 38.5%, *P* < 0.001), while the *KRAS* mutation (17.5% vs. 7.3%, *P* < 0.001) and *MET* alteration rates (6.5% vs. 4.5%, *P* < 0.001) were significantly higher in men than in women. There was no significant difference in the frequency of *BRAF* mutation, *RET* rearrangement, *ROS1* rearrangement, *HER2* alteration, and *ALK* alteration observed between women and men.

### Correlations between genomic alterations and age

**Figure 2:**
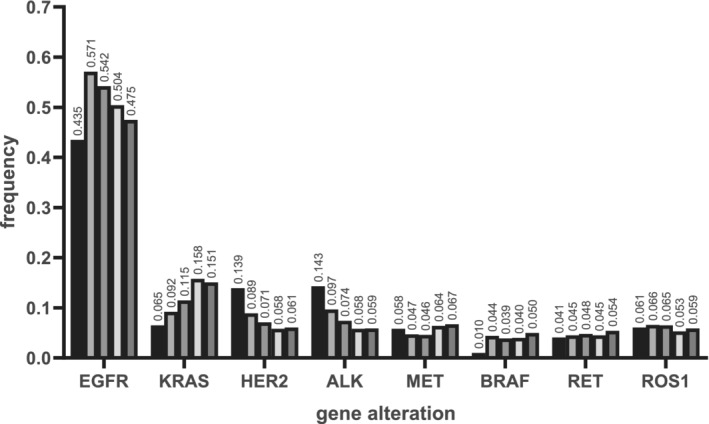
The frequency of selected gene mutations in Chinese patients with non‐small cell lung cancer (NSCLC) among different age groups. *EGFR*, *EGFR* mutations; *KRAS*, *KRAS* mutations; *HER2*, *HER2* alterations; *ALK*, *ALK* alterations; *MET*, *MET* alterations; *BRAF*, *BRAF* alterations; *RET*, *RET* rearrangements; *ROS1*, *ROS1* rearrangements. (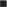) <40, (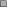) 40–50, (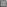) 50–60, (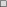) 60–70, (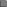) >70.

The mutation rates in different age groups are shown in Fig 2. We found that the *EGFR* mutation rate tended to decrease with age in the group aged >40 years, while the *KRAS* mutation rate tended to increase with age. The *BRAF* mutation rate was 1.0% in the group aged <40 years and approximately 4% in the groups aged >40 years. The *HER2* mutation (13.9% vs. 6.7%, *P* < 0.001) and *ALK* alteration rates (14.3% vs. 6.9%, *P* < 0.001) were significantly higher in the group aged <40 years than in groups aged 40 years or older. The rates of *MET* alteration, *ROS1* and *RET* rearrangement were not significantly different between the group aged <40 years and the group aged 40 years or older.

## Discussion

NGS technology is now widely used to identify the driver genes of NSCLC with resulting data providing a driver gene profile in Chinese patients with NSCLC. Using this technology enables clinicians to make precise clinical decisions. As 68.5% of patients harboring driver genes can receive matched target agents, it is important to carry out NGS, as in addition to typical *EGFR* mutations, other genomic alterations can also be focused upon.

The frequency of *EGFR* mutations, *KRAS* mutations, *HER2* alterations, *ROS1* rearrangements, *RET* rearrangements, *BRAF* mutations and *MET* alterations in this study was consistent with that reported previously in a study in Asian patients.^4^, ^5^ Compared with the Western population, the Chinese patients in this study were found to have a higher frequency of *EGFR* mutation, but a lower frequency of *KRAS* mutation.^6^ It has been previously reported that *NTRK* rearrangements have been found to occur in 0.2% of patients with NSCLC in the Western population,^7^ and the frequency of *NTRK* rearrangements was 0.59% in this study.

*EGFR* mutations include typical and atypical *EGFR* mutations. With the widespread use of NGS, more and more atypical *EGFR* mutations can be detected. In clinical practice, women patients who do not smoke are more likely to be recommended for NGS. In addition, NGS could identify more atypical mutations, and might lead to a higher prevalence of *EGFR* mutations in female patients. Patients with atypical *EGFR* mutations have been reported to have variable efficacy to EGFR TKIs. As atypical *EGFR* mutations account for about 20% of all detected *EGFR* mutations, and 17.7% of patients harbor complex *EGFR* mutations, efficacy of EGFR TKIs in patients with different atypical and complex EGFR mutations need to be further researched. The *EGFR* mutation rate in our study tended to decrease with age, apart from in the group aged <40 years, which is consistent with previously reported data,^4^ which implies that patients in the group aged 40–50 had the highest *EGFR* mutation rate.

*HER2* mutations in NSCLC are dominated by in‐frame insertions in exon 20 of the *HER2* kinase domain.^8^ *HER2* mutation is found in 2%–4% of lung cancer patients.^4^, ^9^ The frequency of *HER2* mutation in this study was 7.0%. The domain structure consists of two ligand binding domains, two cysteine‐rich domains, a short transmembrane domain, a tyrosine kinase domain, and a carboxy terminal tail.^10^ The *HER2* extracellular domain mutants were activated by two distinct mechanisms, characterized by elevated C‐terminal tail phosphorylation, or by covalent dimerization mediated by intermolecular disulfide bond formation.^11^ Different *HER2* variants exhibit divergent sensitivities to anti‐*HER2* treatments. Afatinib, pyrotinib and poziotinib are regarded as HER2‐TKIs. Certain variants, G778_P780dup and G776delinsVC, derive sustained clinical benefits from afatinib, whereas the predominant variant, A772_G775dupYVMA, is resistant to most anti‐*HER2* treatments.^12^ In one study, chemotherapy was found to achieve better outcomes than afatinib for YVMA insertions.^13^ Further clinical trials involving variable *HER2* mutations are required.

Although fluorescence in situ hybridization (FISH) has been established as a gold standard method in the detection of *ALK* and *ROS1* rearrangement, NGS is also a reliable technique.^14^, ^15^ In addition, NGS has been reported to identify different types of *ALK* fusions and *ALK* mutations that mediate resistance to *ALK* inhibitors.^16^, ^17^ The *ALK* rearrangement rate in this study was consistent with that observed in prior studies.^5^

Dysregulation of the *MET* pathway in lung cancer occurs via a variety of mechanisms including gene mutation, amplification, rearrangement, and protein overexpression.^18^ *MET* exon 14 encodes part of the juxtamembrane domain. Juxtamenbrane domain mutations that disrupt splice sites flanking *MET* exon 14 result in *MET* exon 14 skipping. The prevalence of *MET* exon 14 skipping mutations was 0.4% in this study, consistent with a previous report in Chinese patients.^4^ *MET* copy‐number gains arise from two distinct processes: polysomy and amplification.^19^ *MET* amplification is thought to be an oncogenic driver. Copy number gains detected via NGS are reported as continuous variables. Determination of the cutoff related to the efficacy of *MET* inhibitors requires further clinical data.

It has been previously reported that *BRAF* mutations have been observed in 2%–4% of patients with NSCLC.^20^ However, the association between *BRAF* mutation status and patient age or sex appears to be less clear.^21^ The frequency of *BRAF* mutation in this study was similar to the frequency reported in other research. Our study showed no significant association between sex and *BRAF* mutation frequency, but there was a lower frequency in the group aged <40 years than in the group aged 40 years or older. *BRAF* mutations can be divided into V600E and non‐V600E. A total of 202 of all patients with *BRAF*‐mutant NSCLC in this study presented with non‐V600E mutations. Vemurafenib monotherapy has been reported to be effective for treating patients with *BRAF* V600‐mutated NSCLC, but not those with *BRAF* non‐V600 mutations.^22^ Therefore, more effort into the treatment of patients with non‐V600E mutation should be made in the future.

This study has a few limitations. First, it was retrospective, and there may have been a patient selection bias. It has been previously reported that adenocarcinoma accounts for approximately 40% of lung cancers, and squamous cell carcinoma 25% to 30% of lung cancers.^23^ In this study, there were 5382 cases (72.9%) of lung adenocarcinoma, and 855 (11.6%) cases of lung squamous cell carcinoma. The frequency of driver gene mutations was found to be much higher in lung adenocarcinoma than in lung squamous cell carcinoma patients. Therefore, it is recommended that NGS is conducted in more patients with lung adenocarcinoma. Second, we did not collect clinical outcome information and were unable to analyze the clinical prognosis of patients with uncommon mutations. Third, we did not analyze the effect of smoking history on the prevalence of driver gene mutations. It has previously been demonstrated that *EGFR* mutation is highly prevalent in lung cancer patients who were never smokers.^24^ Therefore, the smoking history in different sex and age groups may affect the prevalence of driver gene mutations.

This study demonstrated that the frequency of different driver genes was diverse in different age‐gender groups. It is anticipated that the results of this study may assist clinicians in clinical decision‐making, and assist government, pharmaceutical researchers and insurance companies in the development of public healthcare strategies.

## Disclosure

The authors have no potential conflicts of interest to disclose.
